# Physical Forces between Humans and How Humans Attract and Repel Each Other Based on Their Social Interactions in an Online World

**DOI:** 10.1371/journal.pone.0133185

**Published:** 2015-07-21

**Authors:** Stefan Thurner, Benedikt Fuchs

**Affiliations:** 1 Section for Science of Complex Systems, Medical University of Vienna, Vienna, Austria; 2 Santa Fe Institute, Santa Fe, New Mexico, United States of America; 3 International Institute for Applied Systems Analysis, Laxenburg, Austria; Tianjin University of Technology, CHINA

## Abstract

Physical interactions between particles are the result of the exchange of gauge bosons. Human interactions are mediated by the exchange of messages, goods, money, promises, hostilities, etc. While in the physical world interactions and their associated forces have immediate dynamical consequences (Newton’s laws) the situation is not clear for human interactions. Here we quantify the relative acceleration between humans who interact through the exchange of messages, goods and hostilities in a massive multiplayer online game. For this game we have complete information about all interactions (exchange events) between about 430,000 players, and about their trajectories (movements) in the metric space of the game universe at any point in time. We use this information to derive “interaction potentials" for communication, trade and attacks and show that they are harmonic in nature. Individuals who exchange messages and trade goods generally attract each other and start to separate immediately after exchange events end. The form of the interaction potential for attacks mirrors the usual “hit-and-run" tactics of aggressive players. By measuring interaction intensities as a function of distance, velocity and acceleration, we show that “forces" between players are directly related to the number of exchange events. We find an approximate power-law decay of the likelihood for interactions as a function of distance, which is in accordance with previous real world empirical work. We show that the obtained potentials can be understood with a simple model assuming an exchange-driven force in combination with a distance-dependent exchange rate.

## Introduction

**Fig 1:**
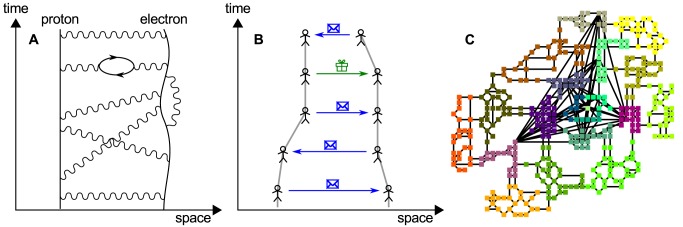
Interactions mediated by exchange of particles. A Electromagnetic interaction between a proton and an electron is mediated by the exchange of virtual photons (after [1]). B Two players in *Pardus* interact by exchanging messages and goods. C Map of the *Pardus* universe. Nodes are sectors (cities), lines between them are connections (streets). Colors represent different regions (countries) [2].

Maybe the most influential concept in physics is the notion of Newton’s laws of motion, which say that in any inertial frame the external forces *F* that act on an object are proportional to its acceleration *a*, or *F* = *m* *a*, where the proportionality constant is the mass of the object. This law is the heart of classical mechanics. In physics there are four forces that are considered to be fundamental, the electromagnetic force, that exists between electrically charged and magnetic objects, the weak force that acts on subatomic particles, the strong force that is responsible for the interactions between quarks, and finally gravitation, that acts between objects that have a mass. The origin of these four forces has been clarified over the 20th century. The current view is that the forces can be understood in terms of exchange of so-called virtual bosons between interacting particles. Electromagnetism is a result of the exchange of photons, see Fig 1A, the weak force rests on the exchange of W- and Z-bosons, and exchange particles of the strong force are the so-called gluons. Gravitation is thought to be mediated by the exchange of (hypothetical) gravitons. By treating virtual exchange particles as excitations of a field the functional form of the interaction potential can be derived [3, 4] from first principles.

In classical physics a force can often be expressed as a negative gradient of a potential *V*(**x**),

$$\begin{array}{c} m\;a=m\frac{d^{2}}{dt^{2}}x=-\nabla V_{}\left(x\right). \end{array}$$ (1)

If a central force is present, meaning that only the distance *r* between the two particles matters, the potential becomes a function of *r*, *V*(**x**) = *V*(*r*), and we get

$$\begin{array}{c} ma=-\frac{d}{dr}[V_{}\left(r\right)+V^{0}\left(r\right)], \end{array}$$ (2)

where *V* ^0^(*r*) is an effective-potential term, which can arise for example by the presence of angular momenta $V^{0}(r)=\frac{L^{2}}{2mr^{2}}$ (in cylindrical coordinates).

Similar to physical interactions, human interactions are to a large extent based on the concept of exchange. The objects that are exchanged can be information, messages, goods, money, presents, promises, aggression, bullets, etc. In Fig 1B we schematically draw the trajectories of two individuals who exchange messages and a gift; their relative distance reduces over time. It is in general not possible to experimentally determine if exchange events between humans generate effective attractive or repulsive forces that influence their relative trajectories. This is due to the lack of simultaneous information on the exchange events and the trajectories of the involved individuals. The existence of potentials causing and influencing the relative motion of humans is not new and has been conjectured in [5]. New technologies in data acquisition and storage are about to change the experimental situation. Data from mobile phone networks, email networks, and several online social networks show that the probability for interaction events decays with distance as an approximate power-law, *P* ∝ *r* ^−*α*^ [6–14], with exponents ranging from *α* = 0.83 [13] to *α* = 2.0 [6–8], see Table 1. Few empirical studies go beyond the analysis of the relation between distance and social dynamics. It was found that humans mostly travel towards others with whom they share a (weak) tie [15]. In [13] human mobility is described as a combination of a periodic daily pattern (from “home” to “work”) and long-distance travels which are influenced by social networks. This model was successfully applied to mobile phone data and the social networks *Brightkite* and *Gowalla* [13]. In a slightly wider context, the role of the number of interaction partners in spacial iterated prisoner’s dilemma games on regular lattices has been shown to have an influence on the size of collaborative clusters [16, 17]. There however the players are static, and do not represent real individuals.

**Table 1:** Power-law exponents obtained from interaction probabilities as a function of distance for various data sets.

Interaction type	*α*	Source
Phone calls	2	[6]
Call duration	2	[7]
Facebook, email, etc.	1.98	[8]
LiveJournal friendship	1.2	[9]
Email	1	[10]
Facebook friendship	1.05	[11]
Brightkite, Foursquare, Gowalla	0.5–1	[12]
Gowalla friendship	0.82	[13]
Brightkite friendship	0.83	[13]
Twitter, Gowalla, Brightkite	0.7	[14]
Messages in *Pardus*	1.3	Fig 2

In this work we study a unique data set containing all interactions between the players of the massive multiplayer online game (MMOG) *Pardus*. In addition we know the players’ exact positions at any point in time. The MMOG has been extensively studied as a human model society, with respect to a wide number of socio-economic aspects, and including a wide spectrum of methodology [2, 18–25]. In [20, 26] entropy based approaches were used. For recent developments on the use of entropy methods in the context of complex networks see [27–30].

*Pardus* has more than 430,000 players who “live” in a virtual environment and interact with each other in a multitude of ways. The game is open ended and players pursue their self-defined goals. Players earn virtual currency through economic activities such as mining raw materials, processing them, or trading. We consider trading between two players as one form of an exchange event; it usually involves the exchange of goods against currency. Players communicate with each other through the exchange of messages via an internal messaging system which is comparable to one-to-one emails. There exist destructive forms of interaction where players attack each other if they are in close proximity. Additional types of interaction, which are not considered in this work include friendship and enmity markings, destruction of equipment, revenge, piracy, and indirect forms of interaction through the formation of groups and governance. For further details on the game see [19, 31]. Note that while communication can happen over large distances, trading (exchange of goods) and attacks require temporal and spatial “locality”. The different interaction types that we study here are labeled by *β* = 1,2,3 for communication, trade, and attack, respectively. For the case of no interaction we use *β* = 0. In Materials and Methods we define the way in which interactions between players are counted.

The game is constrained to a 2 dimensional virtual universe that is partitioned into 400 so-called “sectors”, which play the role of cities. Sectors are connected by 1,064 local and 77 long-range connections (streets). A map of the universe is shown in Fig 1C. Movement is not for free. Traveling long-distance connections costs more than short moves. Travel can be fast but it takes time; traversing the entire universe needs about three days. We define the distance between two sectors as one “step” (network distance 1) if they are directly connected by a local or a long-range connection. For sectors that are not directly connected, we define their respective distance as their network-, or Dijkstra distance. The sectors are formally embedded into a twodimensional Euclidean space as shown in Fig 1C. However the Euclidean length of the connections has no consequence for the players. Therefore, the effective distance inside the game universe is the Dijkstra distance and not the Euclidean distance. As a side note, these two metrics are strongly correlated, with a Pearson’s correlation coefficient of *ρ* = 0.754 for the distances between all pairs of sectors. The network of sectors has a diameter of 27 steps, see also [2, 31]. Given the metric of the Dijkstra distance we can now not only talk about the position *x*_*i*_(*t*) of player *i* at time *t*, but also about relative distances *r*_*ij*_(*t*) of players *i* and *j*, their relative velocities *v*_*ij*_(*t*), and accelerations *a*_*ij*_(*t*), at any given time *t*. For the definitions of *r*_*ij*_, *v*_*ij*_, and *y*_*ij*_, see Materials and Methods. Time is measured in days.

We can now address a series of questions of how social interactions between humans influence their relative motion. In particular, do interactions predominantly happen locally? Or in other words, what is the interaction density as a function of distance? Is it possible to derive interaction-specific potentials from Eq (2)? How is interaction strength related to the relative motion of players, and does motion change after interactions have taken place? Finally, we try to understand the observed interaction potentials by the help of a simple model.

## Results

### Locality of interactions

**Fig 2:**
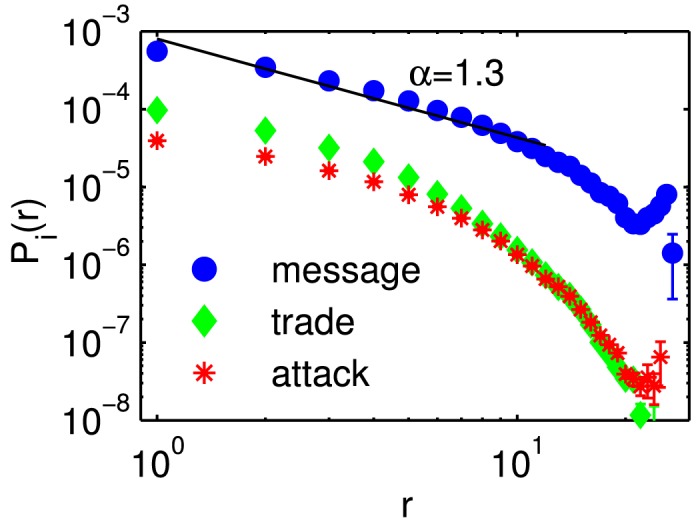
Probability of interactions of type *β*, $P_{i}^{\beta }$ as a function of the distance *r* between players. An approximate power law exponent for communication events is found to be ∼ −1.3 (black line).

We define the probability $P_{i}^{\beta }(r)$ that player *i* interacts with any other player *j* who is at a distance *r* = *r*_*ij*_ away from *i*, within a unit time interval. $P_{i}^{\beta }(r)$ is shown as a function of *r* in Fig 2. Even though the distributions are clearly not power-law, if an approximate power-law exponent was fitted for *β* = 1 (communication). We obtain a slope of ∼ 1.3. In Table 1 this exponent is compared to those found in previous works. For trade and attack, the probability for an interaction decays faster than a power-law. The stronger decay for trade or an attack can be explained by the fact that for these interactions players need to reduce their relative distance to zero within a 24h time window. For *r* > 20 we see a finite size effect that is due to the finite extension of the universe.

### Interaction potentials of social interactions

To obtain the interaction potentials *V* ^*β*^(*r*) it becomes necessary to integrate Eq (2). Since we are working with discrete variables, we set *dr* = 1 and the integration reduces to a simple sum over distances. Assuming that *m* = 1 in Eq (2), the interaction specific potential *V* ^*β*^(*r*) is then simply obtained by

$$\begin{array}{c} V^{\beta }\left(r\right)=-\sum_{r^{\prime }=1}^{r}a^{\beta }\left(r^{\prime }\right)-a^{0}\left(r^{\prime }\right). \end{array}$$ (3)

Here we assume that *V* ^0^(*r*) is caused by the random background motion of players on the finite network of sectors. The relative motion of those players that do not interact with each other (*V*(*r*) = 0) is therefore governed by *V* ^0^(*r*). To estimate *V* ^0^(*r*) in Eq (2) we set *V*(*r*) = 0 and solve for $V^{0}(r)=\sum_{r^{\prime }=0}^{r}a^{0}(r^{\prime })$, where *a* ^*β* = 0^ means acceleration between non-interacting pairs. The resulting unit of *V* is steps^2^/days^2^. Starting the sum at *r*′ = 1 sets the reference point *V* ^*β*^(0) = 0.

**Fig 3:**
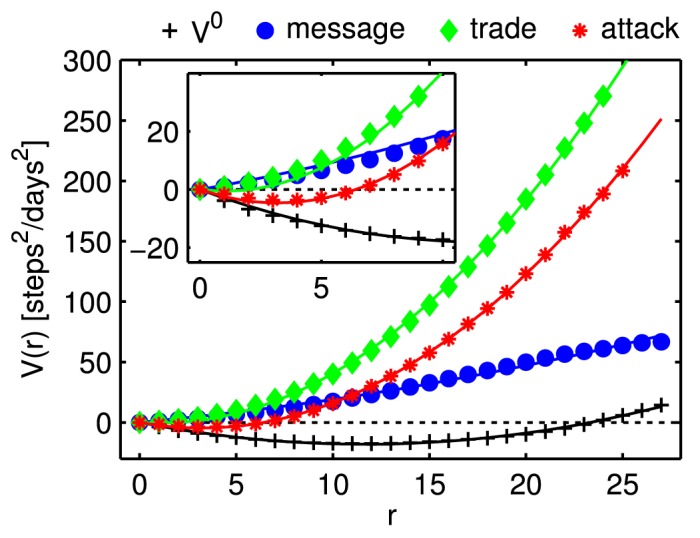
Interaction-specific potentials for messages, trade, and attacks, computed from Eq (3). Solid lines are least-squares fits to a harmonic potential as defined in Eq (4). *V* ^0^ is a result of the background motion of non-interacting pairs of players. The inset is a blow-up for small distances. Clearly, the potential for attack shows a minimum at *r* ^attack^ ∼ 3. For all fits (lines) the explained variance is *R* ^2^ > 0.99.

The resulting potentials for the three interaction types are shown in Fig 3. They can be well approximated by a harmonic and linear potential,

$$\begin{array}{c} V^{\beta }\left(r\right)=\kappa^{\beta }r^{2}-b^{\beta }r, \end{array}$$ (4)

where *κ* ^*β*^ is the respective “force constant”. The corresponding equilibrium distance is at $r_{m}^{\beta }=\frac{b^{\beta }}{2\kappa^{\beta }}$. Potentials increase with distance without signs of saturation. For communication this is consistent with the real-world observation that “(…) the effect friends have on our movement grows with their distance from us” [13]. For trades and attacks there is the simple explanation that players need to reduce their distance to zero at one instance so that the interaction is possible.

We use least-square fits to quantify the parameters in Eq (4). For non-interacting players (*V* ^0^) we find *κ* ^0^ = 0.133 [0.130;0.136], where the intervals give the 95% confidence intervals of the fit. *b* ^0^ = 3.1 [3.0;3.2], corresponding to an equilibrium distance of $r_{m}^{0}=11.7$ steps, which is close to the average distance of any two randomly selected players (12.1 steps). For trade we find *κ* ^trade^ = 0.52 [0.50;0.53], *b* ^trade^ = 1.1 [0.9;1.3], and $r_{m}^{\text{trade}}=1.1$. For attacks we get *κ* ^attack^ = 0.453 [0.447;0.458], *b* ^attack^ = 2.9 [2.8;3.0], and $r_{m}^{\text{attack}}=3.2$. *V* ^attack^ is repulsive for *r* < 3, which reflects a common “hit-and-run” strategy of players. Finally, for messages we have *κ* ^message^ = 0.04 [0.03;0.05], and *b* ^message^ = −1.5 [−1.7;−1.3]. It is obvious from Fig 3 that *V* ^message^ is mainly dominated by the linear term.

### Interaction strength and relative motion

**Fig 4:**
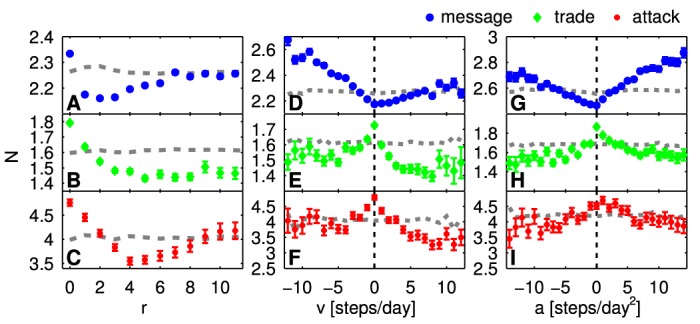
Average number of (uni-directional) interactions per day as a function of *r* ^*β*^, *v* ^*β*^, and *a* ^*β*^. Errorbars are standard deviations of the mean. The results for randomized data are shown with dashed gray lines. Clearly, interaction intensity is strongest for players in the same sector (*r* = 0). For trade and attack, stationary players (with velocity and acceleration zero) interact the most. For messages, interaction is most intense for players who move towards each other but slow down.

We analyze the average number *N* ^*β*^ of messages sent, trades performed, and attacks carried out between the players. Only pairs of interacting players are taken into account. Fig 4, panels A B C show the number of interactions *N* ^*β*^ as a function of distance *r* ^*β*^, respectively. The gray lines indicate the level obtained from shuffled data (see Materials and Methods) which serves as a baseline level. In A we see that the number of exchanged messages is strongly over-represented (above baseline) at zero distance. For all other distances messages are under-represented, reaching the baseline level for large distances. The minimum of messages is found at a distance of 2. Trades are over-represented for distances 0 and 1 (B); attacks for distances 0, 1, and 2 (C). For attacks a clear minimum is reached at distance 4. Panels D E F show the number of interactions *N* ^*β*^ as a function of velocity *v* ^*β*^. It is clear from D that the higher the velocity toward each other the higher is the number of messages. Small positive velocities (away from each other) are slightly underrepresented and approach the baseline for large velocities. The situation is different for trade and attack. Both positive and negative velocities are under-represented (positive ones slightly more). For trade (E) only zero velocity is over-represented. For attacks (F) absolute values of zero and 1 are over-represented. This clearly shows the needed stationarity for trade and attack. Finally, panels G H I display the number of interactions *N* ^*β*^ as a function of acceleration *a* ^*β*^. G shows that the number of sent messages increases from a minimum at *a* = 0 with the radial acceleration: the highest number of messages is exchanged between players who move towards each other, but slow down. For trades and attacks, accelerations close to zero are over-represented, while the baseline is approached for large accelerations. Both are skewed towards positive values of the acceleration.

**Fig 5:**
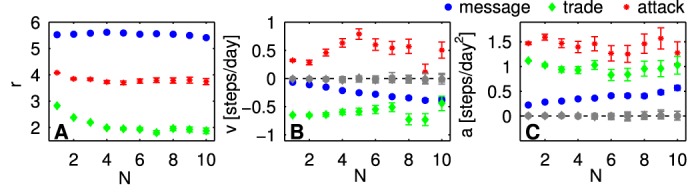
Average *r* ^*β*^, *v* ^*β*^, and *a* ^*β*^ as a function of the number of interactions *N* ^*β*^. Errorbars are standard deviations of the mean. Randomized data are shown with gray symbols. For the randomized data *r* ^*β*^ ∼ 12.1 for all interaction types and independent of the interaction strength (not shown). Characteristic distances are relatively constant in *N* ^*β*^, the characteristic convergence speed for communication increases with the number of messages. The separation speed for attacks increases up to *N* ^attack^ ∼ 4 and remains constant afterward. Acceleration in positive direction, i.e. slowing of convergence for messages increases with *N* ^message^.

In Fig 5 we show distance, velocity and acceleration as a function of the number of interactions. Randomized data is shown by gray symbols. In panel A it is visible that the average distance for messages is about 6.5, for trade about 2, and just below 4 for attacks. These characteristic distances depend relatively little on the number of interactions *N* ^*β*^. The relative large distance for attacks might reflect a “safety” distance. For the randomized data the characteristic distance is *r* ∼ 12.1 for all interaction types and independent of the number of interactions. In Fig 5B it is seen that for trade there is a typical constant convergence speed of ∼ 0.6 irrespective of interaction counts. For messages the characteristic convergence speed increases slightly with the number of interactions. The positive characteristic separation speed of attacks is quite pronounced, and increases with the number of attacks until a plateau is reached from about *N* ^attack^ ∼ 4 on. Characteristic acceleration values for trade and attack are constant in *N* ^*β*^, for communication there is a slight increase of acceleration with *N* ^message^.

### Relative motion before and after interactions

**Fig 6:**
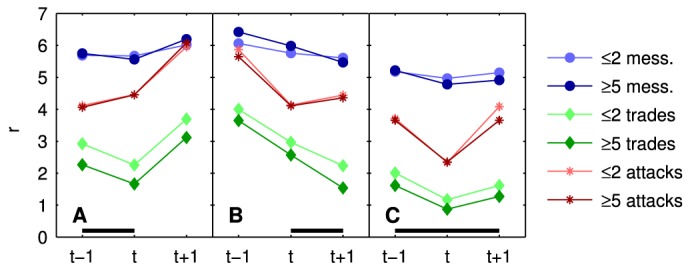
Relative distance *r* ^*β*^ of players before and after interactions. The black line indicates the time interval where interactions take place. A the players interact on the first day, but not on the second, B the players interact on the second day, but not on the first, C the players interact on both days.

Finally we study the effects on characteristic distances right before and after interaction events. In Fig 6 we look at characteristic distances *r* ^*β*^ at three consecutive time points. Time windows where interactions happen are indicated by a black bar. Panel 6 A shows how distances change after a period where interaction(s) occurred. It is clearly visible that when players cease to interact *r* ^*β*^ immediately increases (at *t*+1), i.e. they move away from each other. The effect is especially pronounced for *β* = 2,3 (trade and attack). There is a slight indication that the higher the number of trades, the closer the interacting players are (light and dark colors), see also Fig 5. From Fig 6B we learn that right before interactions take place, players approach each other (from *t*−1 to *t*). This effect can be understood in the following way: If two players are closer to each other than the expected distance for a random pair of players, and assuming that they move independently and randomly, there is an increased likelihood that they will be farther apart on the next day. Similarly there is an increased likelihood that they have been farther apart one day earlier. This effect constitutes the repulsion at $r<r_{m}^{0}$ captured by *V* ^0^. Since *P*_*i*_(*r*) (Fig 2) causes interacting players to be close to each other, i.e. mostly closer than $r_{m}^{0}$, they move towards each other before an interaction. The strongest effect is seen for attacks, for which the beginning separation after the attack is also visible at *t*+1. For attacks we clearly identify a “hit-and-run” tactics where before the attack the players move towards each other (attacker closes in on victim). Right after the attack the attacker moves away from the victim, see panel A. In Fig 6C we see that players who communicate with each other for a more extended period of time (2 days) are closer than those who begin or end a communication, (compare to *r* ^message^ in panels A and B). Their distance remains approximately constant over the three days. The same observations hold for trades. For attacks, again the “hit-and-run” tactics is visible.

### Model

We try to understand the observed interaction potential for messages with the use of a simple model. The basic idea is to model a pair of random walkers *i* and *j* on the *Pardus* universe network Fig 1C. At every timestep *i* sends a message to *j* according to the distance-specific interaction probability $P_{i}^{\beta =\text{message}}(r)$, shown in Fig 2). Both players move to a randomly chosen new sector, which is *d* steps from their current position. *d* is sampled independently for each player from the empirical jump-distance distribution in the game, 𝓟(*d*), see Materials and Methods. If an interaction took place at the current timestep *t*, the player who initiated the interaction (sent the message) moves one step towards the other player along one of the shortest paths. If an interaction took place at the previous timestep *t*−1, the player who initiated the interaction moves two steps towards the other player along (one of) the shortest path(s). If both players have initiated an interaction, both move towards each other independently, each one step at the timestep they initiated the interaction and two steps in the timestep after the interaction. Note that it is highly unlikely that both players initiate an interaction at the same time, since $P_{i}^{\beta =\text{message}}(r)<10^{-3}$ for all *r*. If *i* and *j* are already within the same sector, they remain there. The procedure is repeated for 5 × 10^8^ days for 20 different random initializations.

**Fig 7:**
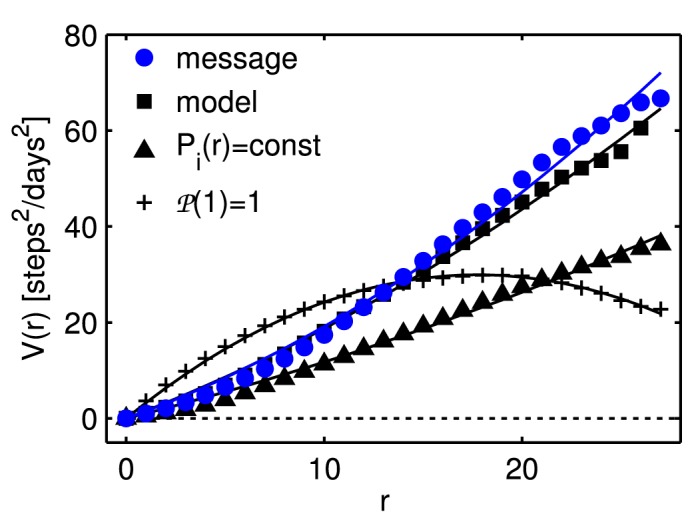
Interaction potential derived from the random walker model. The interaction potential derived from the random walker model (squares) reproduces to a large extent the empirical potential for messages (blue circles). The model uses the actual topology of the universe and assumes an attractive acceleration that is conditional on interaction but independent of the distance. Triangles show the situation where the distance-dependent interaction probabilities are set to be constant, (+) represent an unrealistic constant jump-distance distribution of one step per time. Lines are least-squares fits to harmonic potentials as in Eq (4).

From the resulting relative movements of the players we derive the potential in exactly the same way as before by using Eq (3). In Fig 7 we see that the model (squares) reproduces the potential to a large extent. The model is further able to explain the motion of players toward each other before an interaction that was mentioned in the context of Fig 6B. Note that the inputs were the topology of the universe, the empirical jump-distance distribution of players, the empirical distance-dependent interaction probability, and an acceleration that does not depend on the distance.

To illustrate the importance of the distance-dependent interaction probability we set *P*_*i*_(*r*) = const, and arrive at a potential that underestimates the empirical one (triangles). The effect of the jump-distance distribution is seen when we set 𝓟(1) = 1, i.e. players always move one step, bigger moves as well as no moves are now forbidden. The resulting potential (+) has no more explanatory power. The same result is obtained when setting *P*_*i*_(*r*) = const, and 𝓟(1) = 1 (not shown). Note that for the case 𝓟(1) = 1, *V* ^0^ in Eq (2) is no longer the one shown in Fig 3.

## Discussion

We present a phenomenological study of a comprehensive data set of literally all social interactions and all trajectories of the inhabitants (players) of the virtual world *Pardus*. The data allows us to investigate if social interactions have an influence on the relative movements of players within the virtual universe. We first focused on the locality of interactions by looking at the probability to interact, as a function of distance between two players. Social exchange intensities are found highest for characters that occupy the same sector. We find that the probability to interact decays with relative distance (very) roughly as a power-law with an exponent of about 1.3. We relate this exponent to previous estimates of power-law exponents in a variety of real world settings [6–14].

We find that relative movement patterns of people in the game can be well understood by approximately harmonic interaction potentials. These potentials vary considerably for different types of social interactions. *V* ^attack^ has a clear minimum at distance of 3.2 steps, which is close to the observed average distance between players attacking each other (slightly below 4). We observe a characteristic distance of players interacting through messages between 5 and 6 steps, and about 2 for those interacting through trade. Since we can not define “kinetic energy” in a meaningful way in the game framework, it is not possible to understand the typical distances as “bound states” of interacting individuals.

Players who exchange messages or trade with each other generally tend to move towards each other; when they stop communicating, they typically move away from each other. This is consistent with earlier studies on real-world data, which showed that positive social ties are attractive [13, 15], and that the attraction increases with distance [13]. Players show a tendency to move towards each other shortly before they interact. The more intensive the communication (number of exchanged messages), the stronger is this effect. For an attack, the attacker usually closes in on her victim before the attack and backs up directly after having carried it out. This “hit-and-run” tactics is clearly reflected in the respective potential.

To understand the origin of the potential for the message exchange, we devise a simple model of random walkers, whose interactions cause an attractive acceleration. The model allows to disentangle several relevant effects such as the distance-dependence of interaction probability, and the jump-distance distribution of walkers. Both are essential for explaining the observed potential. The main message learned from the model is that it is possible to understand the distance-dependence of inter-human forces from the distance-dependence of interaction probabilities. The acceleration following individual interactions can be uniform and independent of relative distance.

## Summary and Conclusion

The most important finding in this work is that social interactions (mediated through exchange processes) lead to a measurable reduction or increase of relative distances and acceleration between people in the game. The type of social interaction turns out to be essential for the details of these “forces”. Even though we believe that we were able to quantitatively characterize these forces there are some shortcomings of the work that should be addressed in future work. The origin of the background potential *V*_0_ is yet to be understood. It is conceivable that an appropriate mean field assumption together with taking the geometry of the “universe” into account could lead to its full understanding. Another area for potential improvement could be the use of alternative distance measures other than the Dijkstra distance that was used here, such as distances mentioned in [32, 33]. The discussion of human interactions and their influence on relative movements in different metrics could be an interesting topic for future work. Even though we have seen in previous work that many social networks in the game are very similar to those observed in the real world, it is necessary to stress that the results from this work might not be directly reflected in the real world. Further research is necessary on other data sets that contain both, information on social interactions and localization of humans simultaneously. Mobile phone data could be potentially used to perform a similar study in a real world scenario.

## Materials and Methods

### Interaction counts

We use $N_{ij}^{\beta }(t)$ for the number of times *i* interacted with *j* in the time interval [*t*, *t*+1[. Time is measured in days, i.e. *t* represents a day. *β* = 1,2,3 specifies the type of interaction, communication, trade, and attack, respectively. For the case of no interaction we use *β* = 0.

### Definition of distance, velocity and acceleration

We denote the position of player *i* at time *t* (measured in days) by *x*_*i*_(*t*). Every position of a player is inside one sector and we define the distance of two players *i* and *j* as

$$\begin{array}{c} r_{ij}\left(t\right)\equiv D\left(x_{i}\left(t\right),x_{j}\left(t\right)\right), \end{array}$$ (5)

where *D*(*x*, *y*) is the Dijkstra distance between sectors *x* and *y*. The players’ (relative radial) velocity *v*_*ij*_(*t*), and acceleration *a*_*ij*_ are

$$\begin{array}{ccc} v_{ij}\left(t\right) & \equiv & D\left(x_{i}\left(t+1\right),x_{j}\left(t+1\right)\right)-D\left(x_{i}\left(t\right),x_{j}\left(t\right)\right),\\ a_{ij}\left(t\right) & \equiv & D\left(x_{i}\left(t+1\right),x_{j}\left(t+1\right)\right)+D\left(x_{i}\left(t-1\right),x_{j}\left(t-1\right)\right)-2D\left(x_{i}\left(t\right),x_{j}\left(t\right)\right), \end{array}$$ (6)

respectively. For every interaction type *β*, the average distance *r* ^*β*^ is the conditional average over all distances between interacting players in the time window [*t*, *t*+1[,

$$\begin{array}{c} r^{\beta }=\langle r_{ij}\left(t\right)|i\;\;\text{interacts}\;\;\text{with}\;\;j\;\;\text{through}\;\;\beta \;\;\text{in}\;\;[t,t+1{\left[\right\rangle }_{\left\{i,j,t\right\}}. \end{array}$$ (7)

The average velocity *v* ^*β*^, and number of interactions *N* ^*β*^ are computed in the same way. The average acceleration as a function of the distance is calculated as the average over all interacting pairs {*i*, *j*} given that *r*_*ij*_(*t*) = *r*,

$$\begin{array}{c} a^{\beta }\left(r\right)\equiv \langle a_{ij}\left(t\right)|i\;\;\text{interacts}\;\text{with}\;j\;\text{through}\;\beta \;\text{in}\;[t-1,t+1{\left[,\;\text{and}\;r_{ij}\left(t\right)=r\right\rangle }_{\left\{i,j,t\right\}}. \end{array}$$ (8)

Negative velocity means motion toward each other. Note that negative acceleration can mean three things: either that players increase speed towards each other, or that they slow down when moving apart, or that they change their direction from moving apart to moving towards each other. Most players interact with several others at the same time and the effect of interactions between a pair of players is potentially disturbed by other interactions and factors. Assuming naive superposition of dyadic interactions by taking averages, random disturbances should cancel out.

### Data

Our data contain all players’ positions at a time resolution of one day over 1,238 consecutive days. Messages, trades, and attacks are recorded with a time resolution of one second. To accurately describe the motion of non-interacting players we exclude inactive players on a day-to-day basis: For all results containing acceleration *a* we only consider players who have performed at least one action between *t* − 1 and *t*+1; for all other results players must have at least performed one action between *t* and *t*+1. Given these requirements we get 3,414,091 data points of 31,496 unique players on 1,237 days. For the one-day time interval we have 3,126,842 occurrences of messages, 358,825 occurrences of trades, and 169,227 occurrences of attacks. For all results regarding *a*, pairs of players who interacted only on one of two consecutive days are wrongly treated as two independent data points by the procedure described above. To correct for this error the standard error of the mean is multiplied by a factor of $\sqrt{2}$ to account for the (at most) two-fold over-estimation of the number of independent data points.

### Randomized data

Data are reshuffled by assigning the positions (*x*_*i*_(*t* − 1),) *x*_*i*_(*t*) and *x*_*i*_(*t*+1) to some other active player *j* at random. This is done separately for every day. This way individual trajectories, population densities, and interaction networks are left intact while the relation between positions and interactions are randomized.

### Jump-distance distribution 𝓟(*d*)

For all players *i* who have at least performed one action between *t* and *t*+1, we measure the jump-distance *d*_*i*_(*t*) ≡ *D*(*x*_*i*_(*t*), *x*_*i*_(*t*+1)). $\hat{𝓟}\left(D(x_{i}(t),x_{i}(t+1))=d\right)$ denotes the empirical probability distribution of these jump-distances. The distribution used in the model, 𝓟(*d*), is derived from the measured probability of travel distances $\hat{𝓟}(d)$ by setting 𝓟(*d*) ≡ 0 for *d* > d*, where d* ≡ min_*x*_ max_*y*_ *D*(*x*, *y*) = 20, and normalizing.

## Supporting Information

S1 Data Data as plotted in Figs 2–7. (ZIP)

## References

[1] Feynman RP. QED: The strange theory of light and matter. Princeton, NJ: Princeton University Press; 1985.

[2] Szell M, Sinatra R, Petri G, Thurner S, Latora V. Understanding mobility in a social petri dish. Sci Rep. 2012;2:457 10.1038/srep00457 22708055 PMC3375635

[3] Feynman RP. Quantum electrodynamics In: Pines D, editor. Advanced book classics. Reading, MA: Addison-Wesley; 1998.

[4] Wilson KG. Confinement of quarks. Phys Rev D. 1974;10:2445–2459. 10.1103/PhysRevD.10.2445

[5] Helbing D, Molnár P. Social force model for pedestrian dynamics. Phys Rev E. 1995;51:4282–4286. 10.1103/PhysRevE.51.4282 9963139

[6] Lambiotte R, Blondel VD, de Kerchove C, Huens E, Prieur C, Smoreda Z, et al Geographical dispersal of mobile communication networks. Physica A. 2008;387(21):5317–5325. 10.1016/j.physa.2008.05.014

[7] Krings G, Calabrese F, Ratti C, Blondel VD. Urban gravity: a model for inter-city telecommunication flows. J Stat Mech. 2009;2009(07):L07003 10.1088/1742-5468/2009/07/L07003

[8] Levy M, Goldenberg J. The gravitational law of social interaction. Physica A. 2014;393(0):418–426. 10.1016/j.physa.2013.08.067

[9] Liben-Nowell D, Novak J, Kumar R, Raghavan P, Tomkins A. Geographic routing in social networks. Proc Natl Acad Sci U S A. 2005;102(33):11623–11628. 10.1073/pnas.0503018102 16081538 PMC1187977

[10] Adamic L, Adar E. How to search a social network. Soc Networks. 2005;27(3):187–203. 10.1016/j.socnet.2005.01.007

[11] Backstrom L, Sun E, Marlow C. Find me if you can: improving geographical prediction with social and spatial proximity. In: Proceedings of the 19th International Conference on World Wide Web. WWW’10. New York, NY, USA: ACM; 2010. p. 61–70.

[12] Scellato S, Noulas A, Lambiotte R, Mascolo C. Socio-spatial properties of online location-based social networks. In: Proceedings of the fifth international AAAI conference on weblogs and social media; 2011. p. 329–336.

[13] Cho E, Myers SA, Leskovec J. Friendship and mobility: user movement in location-based social networks. In: Proceedings of the 17th ACM SIGKDD international conference on knowledge discovery and data mining. KDD’11. New York, NY, USA: ACM; 2011. p. 1082–1090.

[14] Grabowicz PA, Ramasco JJ, Gonçalves B, Eguíluz VM. Entangling mobility and interactions in social media. PLoS ONE. 2014;9(3):e92196 10.1371/journal.pone.0092196 24651657 PMC3961345

[15] Phithakkitnukoon S, Smoreda Z, Olivier P. Socio-geography of human mobility: A study using longitudinal mobile phone data. PLoS ONE. 2012;7(6):e39253 10.1371/journal.pone.0039253 22761748 PMC3386290

[16] Wang J, Xia C, Wang Y, Ding S, Sun J. Spatial prisoner’s dilemma games with increasing size of the interaction neighborhood on regular lattices. Chin Sci Bull. 2012;57(7):724–728. 10.1007/s11434-011-4890-4

[17] Zhu C, Sun S, Wang L, Ding S, Wang J, Xia C. Promotion of cooperation due to diversity of players in the spatial public goods game with increasing neighborhood size. Physica A. 2014;406:145–154. 10.1016/j.physa.2014.03.035

[18] Szell M, Thurner S. Measuring social dynamics in a massive multiplayer online game. Soc Networks. 2010 9;32:313–329. 10.1016/j.socnet.2010.06.001

[19] Szell M, Lambiotte R, Thurner S. Multirelational organization of large-scale social networks in an online world. Proc Natl Acad Sci U S A. 2010;107(31):13636–13641. 10.1073/pnas.1004008107 20643965 PMC2922277

[20] Thurner S, Szell M, Sinatra R. Emergence of good conduct, scaling and Zipf laws in human behavioral sequences in an online world. PLoS ONE. 2012;7(1):e29796 10.1371/journal.pone.0029796 22253784 PMC3257232

[21] Szell M, Thurner S. How women organize social networks different from men. Sci Rep. 2013;3:1214 10.1038/srep01214 23393616 PMC3566601

[22] Klimek P, Thurner S. Triadic closure dynamics drives scaling laws in social multiplex networks. New J Phys. 2013;15:063008 10.1088/1367-2630/15/6/063008

[23] Corominas-Murtra B, Fuchs B, Thurner S. Detection of the elite structure in a virtual multiplex social system by means of a generalized k-core. PLoS ONE. 2014;9:e112606 10.1371/journal.pone.0112606 25541957 PMC4277282

[24] Fuchs B, Thurner S. Behavioral and network origins of wealth inequality: insights from a virtual world. PLoS ONE. 2014 8;9(8):e103503 10.1371/journal.pone.0103503 25153072 PMC4143195

[25] Fuchs B, Sornette D, Thurner S. Fractal multi-level organisation of human groups in a virtual world. Sci Rep. 2014;4:6526 10.1038/srep06526 25283998 PMC4185380

[26] Sinatra R, Szell M. Entropy and the predictability of online life. Entropy. 2014;16(1):543–556. 10.3390/e16010543

[27] Thurner S, Tsallis C. Nonextensive aspects of self-organized, scale-free, gas-like networks. Europhys Lett. 2005;72:197–203. 10.1209/epl/i2005-10221-1

[28] Cao S, Dehmer M, Shi Y. Extremality of degree-based graph entropies. Inf Sci. 2014;278:22–33. 10.1016/j.ins.2014.03.133

[29] Kraus V, Dehmer M, Schutte M. On sphere-regular graphs and the extremality of information-theoretic network measures. MATCH Commun Math Comput Chem. 2013;70:885–900.

[30] Chen Z, Dehmer M, Shi Y. A note on distance-based graph entropies. Entropy. 2014;16(10):5416–5427. 10.3390/e16105416

[31] Bayer & Szell OG. Pardus: free browser game set in space. 2003–2015. Available: www.pardus.at

[32] Randic M. DMAX-matrix of dominant distances in a graph. MATCH Commun Math Comput Chem. 2013;70:221–238.

[33] Xu K, Liu M, Das KC, Gutman I, Furtula B. A Survey on graphs extremal with respect to distance-based topological indices. MATCH Commun Math Comput Chem. 2014;71:461–508.

